# Impact of cancer diagnosis and treatment: a qualitative analysis of strains, resources and coping strategies among elderly patients in a rural setting in Ghana

**DOI:** 10.1186/s12877-023-04248-8

**Published:** 2023-09-05

**Authors:** Adwoa Bemah Boamah Mensah, Maurice Mikare, Kofi Boamah Mensah, Joshua Okyere, Er-Menan Amaniampong, Abena Agyekum Poku, Felix Apiribu, Joe-Nat Clegg Lamptey

**Affiliations:** 1 School of Nursing & Midwifery, College of Health Sciences, Private Mail bag, Kwame Nkrumah University of Science and Technology, University Post Office, Kumasi, Ghana; 2 Nursing and Midwifery Training College - Zuarungu, P. O. Box 660, Bolgatanga, Ghana; 3 Department of Pharmacy Practice, Faculty of Pharmacy and Pharmaceutical Sciences, College of Health Sciences, Kwame Nkrumah University of Science and Technology, University Post Office, Private Mail bag, Kumasi, Ghana; 4 Department of Population and Health, University of Cape Coast, University Post Office, Cape Coast, Ghana; 5 Department of Sociology and Social Work, Faculty of Social Sciences, College of Humanities and Social Sciences, Kwame Nkrumah University of Science and Technology, University Post Office, Private Mail bag, Kumasi, Ghana; 6 Department of Komfo Anokye Teaching Hospital, P. O. Box, 1934, Kumasi, Ghana; 7 Department of Surgery, University of Ghana Medical School, University of Ghana, Accra, Ghana; 8 Korle-Bu Teaching Hospital, Accra, Ghana

**Keywords:** Cancer, Coping strategies, Elderly, Resources, Strain, Qualitative research, Ghana

## Abstract

**Background:**

Rurality is fraught with numerous difficulties including a lack of advanced health facilities to provide health services, and an absence of specialist cancer services, and qualified personnel, among others. These factors exacerbate the challenges of elderly patients diagnosed with cancer and further pose limitations to activities/instrumental activities of daily living. Yet, there is limited scholarship on the strains that affect elderly patients diagnosed with cancer and the resources that helps them to overcome them. This study explores the strains, resources, and coping strategies of elderly patients diagnosed with cancer and undergoing treatment in rural Ghana.

**Methods:**

An exploratory, descriptive qualitative design was adopted. Purposive sampling was used to recruit 20 individuals to participate in in-depth interviews. The collected data was analysed inductively using Collaizi’s framework. QSR NVivo-12 was used in managing the data.

**Results:**

The results were grouped into two main categories, namely: strains and resources. Within the category of strains, three main themes with their corresponding sub-themes emerged: cancer-related strains (systemic side effects from treatment, altered physical appearance and body image, and experience of pain), elderly strains (altered functional ability, limited social interactions and participation, psycho-emotional reactions, limited/restricted economic participation, and financial strains), and health system strains (negative attitude and insensitive communication, delay in diagnosis, lack of geriatric oncology care, lack of community-based specialist cancer centre and long travel distance to access care, and limited availability of essential cancer medicines and other radiations services). Four types of resources were available to cancer patients: personal resources, family resources, community resources, and healthcare systems resources.

**Conclusion:**

In conclusion, elderly patients diagnosed with cancer experience physical, economic, psychological, and emotional strains that threaten their health and well-being. However, they are able to leverage family, community, and health system-related resources to navigate through the strains. There is, therefore, a need to expand advanced health facilities with geriatric oncology units and specialists to improve access to cancer care in rural areas. The government needs to assist elderly persons with costs associated with their diagnosis and treatment through the expansion of the National Health Insurance Scheme to include this as part of the benefits package.

**Supplementary Information:**

The online version contains supplementary material available at 10.1186/s12877-023-04248-8.

## Background

The global aging population is increasing exponentially, and is expected to reach 426 million by 2050, with more than one in every five persons estimated to be 60 years or older by 2050 [1, 2]. These extra years are predominantly characterised by poor health such as increased susceptibility to cancer, due to degenerative changes and sedentary life that makes older adults relatively physically inactive [1–3]. For instance, by 2035, the number of cancer cases is predicted to increase by approximately 144% among the elderly in developing regions [4]. Ghana is a sub-Saharan Africa (SSA) country on the Western coast of Africa with a population of nearly two million (1,991,736) aged 60 years and older, comprising 1,129,906 (56.7%) females and 861,830 (43.3%) males [5] who are at risk of developing cancer. In 2020 alone, 15,802 persons died of cancer in Ghana with approximately 8,399 older persons being diagnosed with the disease [6]. A narrative review on cancer control in Ghana indicates that 80% of elderly with cancer were living in rural areas and were commonly diagnosed at an advanced stage (stage III/IV) of the disease [7], many months (8–15 months) after first noticing a sign or symptom of the disease [8]. This statistical evidence prospectively suggests that Ghana could experience a rapid increase and deepened cancer-related health and economic burdens or needs among its older adult population [9, 10], creating challenges for elderly cancer patients. According to the 2021 Annual Reports of the Tamale Teaching Hospital, an average of 50 patients, 30 of which are elderly patients, seek cancer care from the facility every month [11].

Literature, however, indicates that the impact of cancer and its treatment for elderly patients living in rural areas is more pronounced and dire which greatly diminishes their health and well-being compared to urban areas [13–15]. These areas are greatly deficient in healthcare access, specialised cancer services, and support resources as found in many developing settings. For instance, in China and Mexico, rural areas have problems accessing cancer care resources and inadequately qualified providers while faced with financial hardships [14], an absence of definitive treatment [13], and increased travel and financial costs to access healthcare [16]. Webb et al. [15] added that aside from the limited qualified personnel, insufficient time and geriatric oncology principles being omitted from formal training and continuous education of health professionals in low-and-middle-income countries (LMICs) exacerbates the impact of cancer and its treatment on patients.

In Ghana, rurality is fraught with numerous difficulties including a lack of advanced health facilities to provide health services, absence of specialist cancer services and qualified personnel, long travel distance and financial costs to access healthcare, financial hardships due to limited access to jobs, resulting in deepened poverty [17–19]. These factors magnify the challenges of elderly patients diagnosed with cancer in rural communities and further pose limitations to activities/instrumental activities of daily living, performance status, and quality of life, reducing their overall survival [16, 20]. Yet, there is limited scholarship on the strains that affect elderly patients diagnosed with cancer and the resources that helps them to overcome these strains in sub-Saharan Africa, especially in Ghana where a substantial proportion of the elderly population is rural-domiciled [12, 13]. This presents a significant gap in what we currently know about the resources that help elderly patients diagnosed with cancer to cope with the strains that they experience. Considering this gap in the literature, this study explores the strains, resources, and coping strategies of elderly patients diagnosed with cancer and undergoing treatment in rural Ghana. Findings from this study provide an insightful basis that could be used to inform practice and policy change to improve health outcomes of elderly patients living with cancer in rural areas.

## Methods

### Study design

An exploratory, descriptive qualitative design was adopted to explore and describe the strains, resources, and coping strategies of elderly patients diagnosed with cancer [14, 15]. Anecdotal evidence suggests that elderly patients living with cancer in rural Ghana might have unique strains, resources, and coping strategies. Yet, studies reporting on the impact of cancer and its treatment on these cohorts of patients are from developed countries and used quantitative study designs [16, 17]. Limited evidence focused on elderly patients living with the disease in sub-Saharan Africa, particularly, the elderly diagnosed with cancer in Ghana, and living in rural communities. The design thus aided the comprehensive understanding of the phenomenon under study.

### Study setting

The study was conducted at the Tamale Teaching Hospital (TTH). This is the only tertiary health facility providing health services to the Northern region of Ghana. This makes the hospital a major medical referral centre for the Savannah, Upper East, North-East, Northern, and Upper West regions [18]. The hospital has a cancer treatment centre, launched in 2021 by the Ministry of Health (MoH) of Ghana () to provide medical oncology services to patients diagnosed with various forms of cancer such as breast, cervical, and prostate cancers [19]. The cancer unit at the Tamale Teaching hospital is managed by a medical oncologist and two Oncology Nurse Specialists. The 2021 TTH Oncology Attendance Register indicates that the average monthly attendance is 50 with about 30 out of this number being elderly [11]. Main services rendered by the clinic are scheduled reviews for chemotherapy and hormonal therapy. Patients needing radiation therapy are referred to the National Center for Radiotherapy and Nuclear Medicine at the Komfo Anokye Teaching Hospital, Kumasi or Korle-Bu Teaching Hospital, Accra. These characteristics made TTH suitable for this study.

### Population ad sampling technique

Elderly diagnosed with and receiving treatment for cancer in the Tamale Metropolis were targeted for the study. Eligibility criteria were: (i) aged 60 years and above; (ii) histologically confirmed malignancy and receiving treatment at TTH at the time of the study, (iii) fluently in the “Twi”, “Dagbani”, “Dagaare” or English language, which were the fluent languages spoken by the researcher. Patients below the age of 60 years and those too ill to communicate were excluded from this study. Participants were purposively recruited into the study [20, 21].

### Recruitment procedure and sample size

The Cancer Unit of TTH was the main recruitment outlet for this study. Ethical approval was granted by the Committee on Human Research, Publication, and Ethics (CHRPE) at the Kwame Nkrumah University of Science and Technology (KNUST). The second author visited the clinic every week, on Tuesdays when cancer patients were scheduled for consultation, to recruit participants. This was assisted by a recruitment link (an oncology nurse specialist at the Oncology unit). Participants whose eligibility was confirmed using the inclusion criteria were engaged in a discussion and were given the study’s information sheet. To ensure that participation was voluntary, eligible participants provided their informed consent within one week and were reminded of their rights to withdraw from the study at any point in time without any repercussions. For the period of recruitment, there were 31 patients, however, only 24 of them were eligible to participate in the study after having been screened with the inclusion criteria. All 24 patients consented to participate in the study. However, at the end of the study we had a sample of 20; these 20 participants provided both in writing (n = 9) and thumbprints (n = 11). Participants’ identities were anonymised using unique study codes. The sample size of 20 was not predetermined but was informed by theoretical data saturation since further interviews yielded no additional insights [22, 23]. As a result, the study reached saturation on the 18th participant after which two more interviews were conducted for confirmation.

### Data collection tool and interviews

Data were collected by the second author between March 2021 and September 2021, through individual, in-depth face-to-face interviews using a semi-structured interview guide (Supplementary File 1). The study’s objectives and existing literature informed the development of the interview guide. Oncology specialists and research supervisor (ABBM) reviewed the interview guide to ensure it captures issues around strains, resources, and coping strategies. The research supervisor is an academic who has decades of experience in qualitative cancer research. Guiding questions were piloted using four elderly patients diagnosed with and receiving treatment for cancer from a private Oncology Centre in the Northern region. This helped to determine the sociocultural suitability, relevance, and clarity of questions, as well as an opportunity to practically correct the initial design and wording of the interview guide. Data from the pilot interviews were excluded from the main findings.

Interview venue, date, and time were scheduled in line with the participants’ preferences. All interviews were conducted in their homes which enhanced their comfort during the interviewing process. At the beginning of each interview, both written and oral consent was sought. Interviews lasted for an average of 50 min. All interviews were conducted in the Dagbani and Dagaare local languages, except three interviews that were done in the Twi local language. Probes were thoughtfully and thoroughly used to deeply explore and understand the strains, resources and coping strategies participants shared. However, throughout the interviews, guiding questions were not added on but rather shaped and rephrased based on emerging data prior to attaining data saturation. Though data saturation was reached on the 18th participant, data collection continued with 19th and 20th participants for confirmatory purposes. Field notes comprising participants’ non-verbal cues (sighs, silence, etc.), concerns, and interviewer reflections were captured.

### Data analysis

An inductive thematic analysis was done using Collaizi’s framework for qualitative analysis. After each day of data collection, the audio data was transcribed verbatim in Twi, Dagbani, and Dagaare languages by the second author (MM). An independent back-back translation process of the anonymised transcripts was carried out by three language experts who were fluent in both local languages. The reason for transcribing on the same day of the interview was to enable the second author (MM) to identify questions that may have been left unanswered during the interview. Subsequently, MM followed up on such transcripts and questions to get detailed accounts from the participants. After the transcription of the data, the transcripts were vetted and proofread by the first, third and fourth authors. When this vetting process was completed, the transcripts were made accessible to the first and fourth author, who performed the initial independent thematic analysis. The transcripts were imported into QSR NVivo-12 for data management and analysis. All 20 transcripts were independently, repeatedly, and thoroughly read by the first and fourth authors to familiarise themselves with the data. Using the ‘nodes’ function in QSR NVivo-12, codes were assigned to the significant recurring phrases or statements in the transcripts. ‘Meanings’ were then formulated from these significant phrases or statements to depict and describe the fundamental reasons for the various contexts of participants’ strains, resources, and coping experiences. Clusters of themes were developed from formulated meanings based on their relationship and similarities. Recurring codes that were similar were categorised to generate themes and sub-themes. Extracts and quotes from the themes and sub-themes generated were used to support the results of the study. We conducted member checking with five participants who confirmed that the findings reflected their perspectives.

### Trustworthiness and researchers’ reflexivity

Recognising the importance of rigour and trustworthiness in qualitative research, the authors worked to ensure confirmability and transferability. The study findings were transferable and confirmable due to the detailed description of the study circumstances and techniques. Member-checking with five of the participants was carried out to establish credibility; this was done one week following the completion of the analysis. None of the participants submitted revisions or raised issues about the content and quality of the interviews in articulating their perspectives. After each interview, field notes were taken and referred to during the analysis, which included the participants’ nonverbal indications, worries, and interviewers’ reflections. Prior to data collection, each author’s prior beliefs, experience, and attitude that might affect the data curation, analysis, and interpretation [24] was assessed. Through this, the first and third authors disclosed extensive experience in clinical practice in oncology and qualitative research on cancer diseases. However, both authors are currently in academia and not working in the study setting, and therefore could not directly influence the study participants, site, methodological approaches, and study findings. The second author who conducted all the interviews is a trained healthcare educator and researcher with substantial experience conducting in-depth interviews. This author does not work in the study settings and had no direct relationship with the participants. None of the authors was related to or had any relations with any of the participants. Adopting these rigorous processes in this study meticulously enhanced the credibility and trustworthiness of the study [25].

### Ethics Approvals

The study conforms to the Declaration of Helsinki. Ethical approval was obtained from the Tamale Teaching Hospital (TTH) and the Committee on Human Research, Publication, and Ethics (CHRPE) at the Kwame Nkrumah University of Science and Technology (KNUST) (reference number: CHRPE/AP/275/21) respectively. To protect the identities of the study participants, the transcripts and audio files were anonymised. An information sheet containing the purpose of the study, procedures, possible risks, and benefits, compensations, who to contact, and affirmation of confidentiality, privacy, and autonomy, was provided to the study participants. The interviewer sought written and oral informed consent from the participants as approved by the CHRPE. This indicated their decision to voluntarily participate in our study after having read and understood the terms of reference. The audio files and transcripts were encrypted to prevent unauthorised persons from having access to the data.

## Results

### Participants’ demographic characteristics

Interviews were conducted among twenty (20) participants, three (3) males and seventeen (17) females. Participants’ ages ranged between 60 and 89 years. The diagnosis of participants was cervical cancer (n = 8), breast cancer (n = 8), hepatocellular carcinoma (n = 1), throat cancer (n = 1), prostate cancer (n = 1), and choriocarcinoma (n = 1). Participants had lived with the disease between 3 months to 3 years. Five (5) participants owned their businesses, five (5) were unemployed, four (4) were farmers, three (3) were petty traders and three (3) were retired public servants. With regards to their religious affiliations, ten (10) participants were Muslims, nine (9) were Christians, and one (1) was a traditionalist. Participants were from varied ethnic groups with a majority from Mole-Dagomba origin (see Supplementary file 2).

### Themes

Table 1 illustrates the themes and sub-themes associated with the strains, resources, and coping strategies of elderly patients diagnosed with cancer. For strains encountered by participants, three major themes were identified: cancer-related strains, elderly strains, and health system strains. Major themes reflecting the resources and coping strategies of participants comprised: personal resources, family resources, community resources, and healthcare system resources.

**Table 1:** Theme and sub-themes

Domain	Theme	Sub-themes
Strains	Cancer-related strains	• Systemic side effects from treatment • Altered physical appearance and body image • Experience of pain
	Elderly strains	• Altered functional ability • Limited social interactions and participation • Psycho-emotional reactions • Limited/restricted economic participation • Financial strains
	Health system strains	• Negative attitude and insensitive communication • Delay in diagnosis • Lack of geriatric oncology care • Lack of community-based specialist cancer center and long travel distance to access care • Limited availability of essential cancer medicines and other radiations services
Resources and coping strategies	Personal resources	• Religious beliefs • Accepting, adapting to one’s condition, and self-reliance
	Family resources	• Spiritual support • Psychological and emotional support • Practical support with activities of daily living/instrumental activities of daily living • Financial resources and support
	Community resources	• Financial and instrumental support from community members
	Healthcare systems resources.	• Follow-up assistance health support/ assistance

### Strains

Challenges posed by cancer diagnosis and treatment to older adult patients were explored in this study. These challenges are conceptualized as strains, three major themes associated with participants’ strains were identified and categorised as: (i) cancer-related strains; (ii) elderly strains; and (iii) health system strains.

### Major theme #1: Cancer-related strains

This major theme describes the challenges experienced by participants as a result of the disease and its related therapies. Sub-themes that emerged were (1) disease and systemic effects from ttreatment, (2) changes in physical appearance (skin change, hair loss, etc.), and (3) restrained functional ability.

#### Systemic side effects from treatment

Findings from the study revealed that participants experienced numerous side effects from the treatments received. This included nausea or vomiting, pain, and cramps, fever, diarrhoea, dizziness, and thirst usually because of the toxicity associated with the treatments they undergo. The symptomatic consequences of treatments received were similarly disclosed by participants in this study. For instance, participants revealed that when they receive chemotherapy or radiotherapy, they become nauseated and experience frequent bowel movements.

*“As I mentioned earlier, the disease has affected me because anytime I go for the treatment (chemotherapy) it makes me experience diarrhoea.” (EP008, female, 80 years, Breast cancer)*.

Other participants added that undergoing anti-cancer therapy affected their eating habits. This was linked to the frequent loss of appetite and vomiting experienced because of the chemotherapy and radiation therapy. This is evidenced in the quotes below:

*“…I lost my appetite following the diagnosis. This got serious with the chemotherapy. For now, I cannot eat at all. I feel nauseated and vomit a lot anytime I receive injections (chemotherapy), and I have completely lost my appetite. The little I eat, I vomit so I do not eat…” (EP006, female, 60 years, cervical cancer)*.

#### Altered physical appearance and body image

This theme describes the experiences of participants relating to the effect of cancer therapy on their physical bodies and appearance. Participants experienced weight loss, skin discolorations, and hair loss. Weight loss was linked to altered eating habits which stem from the side effects of treatment such as loss of appetite, nausea, and vomiting, coupled with diarrhoea. Participants described themselves as looking ‘lean’ when they compared their current weight to their previous weight. This is illustrated in the responses below:

*“I have lost weight. Prior to the treatment, I was not like this. I have grown too lean due to the vomiting and sometimes diarrhoea which occurs when I take the injections (chemotherapy) It has affected the way I look and am not happy about it.” (EP010, female, 68 years, cervical cancer)*.

Additional findings as shared by participants specified that undergoing chemotherapy led to hair loss and discoloration of skin and nails. Participants cited darkened skin and nail beds and related these colour changes to the toxicity of the cancer medications. These are illustrated in the expressions below:

*“…see my head, all my hair is gone, also my nails and my skin colour are becoming dark. Ah! but it was not there, it is when I started coming here [hospital] that all these started so it is the treatment that is causing it.…” (EP002, female, 65 years, cervical cancer).*

#### Experience of pain

In this study, pain was a common feature that characterised the development of the diseases and the exposure to anti-cancer medications. The pain was typically described as burning sensations and abdominal contractions. The passion and emotions with which participants described the pains they experience inform the extent of the pain they experienced. One of the participants, an 80-year-old woman suffering from breast cancer quizzed while narrating her ordeal:

*“…the pain associated with this disease is not small, so how will this end?” (EP008, female, 80 years, Breast cancer)*.

To the participants, the pain became worse after they had received treatments:

*“Another thing that bothered me so much was the pain I was going through. The pain was so severe that I couldn’t even bear. When I swallow saliva, it was very painful…” (EP004, Male, 69 years, Throat cancer)*.

*I have been having some burning sensations over my entire body…When I take the medicine…, the medicine also causes severe abdominal pain. It is very discomforting…” (EP003, female, 64 years, cervical cancer)*.

### Major theme # 2: Elderly strains

This theme describes various physical, psycho-emotional, economic, and social difficulties posed to the participants due to their cancer conditions and associated treatments.

#### Altered functional ability

Elderly’ ability to function daily was one of the key areas they faced challenges due to their cancer disease and treatment side effects. Daily functioning here implies their ability to perform activities of daily living (ADLs). These ADLs are categorised into basic and instrumental activities of daily living (IADLs). While personal care activities including bathing, washing, dressing, and feeding remain basic ADLs, other activities such as performance of house chores, meal preparations, and grocery shopping are considered as IADLs. Participants in this study commonly shared limitations in their activities of daily living, affecting their ability to live independently. The ability to feed, become mobile, and groom one’s self, were major areas of daily living activities that participants reported facing difficulties in performing. The expressions below illustrate participant’s constraints with ADLs:

*“As you see me laying here now, I cannot bathe myself, I cannot groom myself and I feel weak when am walking. Things were not like this before the diagnosis. The last time I walked to the urinary myself, I nearly fell so I have been advised not to walk there alone.” (EP006, female, 60 years, cervical cancer)*.

*“The treatment I was given in Tamale has further made me weak and walking has become more difficult. When I walk small, I have to sit down to rest, it is always like this anything I take the chemotherapy” (EP005, female, 64 years, Breast cancer)*.

An 85-year-old breast cancer patient further confirmed how being able to perform activities of daily living is crucial to one’s independence. She explained:

*“Oh…it [the treatment] has really affected me because I cannot lift my hands due to the pain in my shoulder after the surgery. And if you cannot lift your hands, how will I be able to bath. So I am not able to bath unless I lie down for them to bath and dress me. .” (EP007, female, 85 years, cervical cancer)*.

Female participants expressed cultural obligations to maintaining their households. These include house chores, meal preparations, and grocery shopping. However, in addition to facing difficulties performing the basic activities of daily living, female participants shared that their ability to perform these instrumental daily living activities was greatly affected. These are illustrated in their responses below:

*“…For cooking too, I am not able to cook. I do not think I could cook at this time with this disease…” (EP016, Female, 63 years, Choriocarcinoma)*.

*“…Also, with this heavy bleeding am not able to cook again but earlier I could so. the disease has affected me. Hmmm…” (EP003, female, 64 years, cervical cancer)*.

#### Limited social interactions and participation

Side effects of treatment with its constrained functional ability made it impossible for the participants to engage in forms of social interactions and to participate in social events such as group or association meetings, funerals, and church services. This failure on their part to attend social events in some instances led to their withdrawal or being ostracised from their groups. This portrays how their disease worsens their social functioning and integration into society. The following quotes reflect these experiences of the participants:

*“Now I cannot visit my friends and children as I use to. I have even stopped going to the pensioners’ association meeting, I use to be active in the association. Even funeral I cannot attend again. I urinate frequently due to the disease and the treatment always makes me weak.” (EP017, male, 73 years, Prostate cancer)*.

*“…and I use to be active in church as well but with the weakness, I experience now I cannot always be in church…I am even scared to interact with people because of the bleeding. I don’t feel comfortable to even mixing with people because those who know you will be asking so many questions…” (EP016, female, 63 years, Choriocarcinoma)*.

#### Psycho-emotional reactions

This theme describes how being diagnosed with cancer affected the psychological and emotional health of participants. Being diagnosed with cancer in later life was characterised by negative stressors that adversely exerted dire consequences on the mental and emotional health of participants such as depression, worry anxiety, fear, and distress. Participants related their psycho-emotional reactions to the news of the diagnosis, long travel to hospitals, increased hospital stays, cost of medical care, side effects of cancer therapy, poor treatment outcomes, uncertainty about their recovery, and fear of death. This is illustrated in the quotes below:

*“…when I was told I had cancer I was so frightened. I thought the world had come to an end. Was so afraid I will die. So honestly, I didn’t feel good at all and up till now, I don’t feel good with this disease. Imagine after all these years of suffering and labouring for me to retire only to meet this disease…So now I am not happy at all… too disturbed emotionally.” (EP016, female, 63 years, Choriocarcinoma)*.

*“When I was told that it was cancer, I was very worried and scared knowing and hearing about cancer. I knew I was in a critical situation which is between life and death…I was scared and anxious and not sure of recovery…” (EP008, female, 80 years, Breast cancer)*.

Some of the participants further expressed worry and anxiety concerning the ability to finance their treatment. This is borne out of the fact that cancer was regarded as a ‘disease of the rich.’ Hence living in a rural area where economic resources are scarce could limit the ability of participants to finance their treatment. This was evident in the narratives of a participant as follows:

*“I had spent so much money moving from one facility to the other to get diagnosed, so when they finally told me I have cancer, I was worried about the money I will use to fund the treatment. I have always known cancer to be a disease of the rich. Living in a village with this disease was a source of worry because I do not have that kind of money to cover the cost of care.” (EP002, female, 65 years, cervical cancer)*.

#### Limited/restricted economic participation

This theme describes the economic and financial hardships faced by participants. Most participants in this study mainly earned their source of livelihood from their informal economic activities and being able to maintain these informal business activities was dependent on their continuous engagement. However, with the advent of their disease, participants argued otherwise. This is because their disease subjected them to deteriorating physical strength since they easily become tired with the least activity done. A 63-year-old female diagnosed with cervical cancer, who was involved in her business trades explained saying:

*“…It has affected my ability to work. I use to buy and sell things but now I can’t do them because of this disease. Others are older than me but they are still doing their business very well so I still believe that all these problems are from this disease.” (EP012, female, 63 years, cervical cancer)*.

A male participant whose informal business activity required him to physically move around to potential customers described how his inability to walk for longer distances limited his ability to market his business to others:

*“I could not walk for long because of my condition, and this has affected my ability to walk for long distances to potential customers since I get tired easily. Due to my condition, I am not able to talk or communicate with people about my business anymore.” (EP004, male*, 69 years, Hepatocellular cancer*)*.

Participants’ inability to engage in their usual economic activities further affected their income generation. Hence, the ‘insufficient monies’ as described by participants were no longer earned. This was indicated by A 70-year-old petty trader diagnosed with breast cancer:

*“Currently I cannot sit by my table to sell which means that the small money I use to get is not also coming again because of this disease.” (EP013, female, 70 years, breast cancer)*.

#### Financial strains

This theme describes the monetary challenges that participants faced because of the costs associated with diagnosing and treating the disease. The participants expressed that despite their enrolment onto the national health insurance scheme (NHIS), they still faced significant financial constraints in respect to cancer management. This was mainly from ancillary health expenditure such as doctor’s consultation and treatment fees, medication, and laboratory investigations. This was typically described by the participants as ‘co-payment/top-up’ Other situations beyond participants’ control further led them to frequently pay out of pocket for drugs and laboratory investigation services to avoid interruptions in their 3-weekly treatment cycles. For instance, not all the cancer drugs and laboratory investigations were listed as insured. There was also frequent shortage of insured cancer drugs, laboratory reagents, and other medical supplies at the hospital necessitating participants to outsource these services from private institution out of pocket. Participants described this as expensive and draining, causing a financial crisis. This is illustrated in the quotes below:

*“.…The treatment is very expensive. I have been spending all my money and all these things [laboratory diagnostics, medications] we do here are not covered by the health insurance. I have to cough out the money, so I do not interrupt the chemotherapy. It is difficult…” (EP009, female, 70 years, cervical cancer)*.

*“The disease and treatment have brought financial hardship on me because the treatment process is costly…Is very difficult to get money lately because I am not working and have no source of regular income. Not all the medicines are under insurance, so I pay for it. Most often, insured drugs are not available when I visit for the injection (chemotherapy) so I have to buy from drug store at a very high price. All the labs I am asked to do are not covered by insurance. I spend so much time every 3 weeks. Truthfully, sometimes I skip the treatment due to money.” (EP014, female, 75 years, breast cancer)*.

Similarly, participants whose cancers were excluded from the NHIS package made out-of-pocket payments for all diagnosis and treatment services, imposing greater financial constraints on them. This led some to sell their life-acquired properties to aid them access treatment. This was affirmed in the response below:

*“My cancer is not supported by the insurance at all. Hence, I had to pay for everything from the diagnosing period. Every lab test I was made to do to confirm the disease was very expensive. Then came the main cancer treatment! Things have not been easy at all. Hardship upon hardship. I had no option but to sell my farm in support of my treatment. The treatment is draining…” (EP017, male, 73 years, Prostate cancer)*.

The cost of medical care was further worsened by other indirect costs such as transportation and feeding during review visits. Due to the geographical location of the cancer center (capital city), most participants traveled long distances from their rural communities to access care on an outpatient basis via appointment. The travel and cost of living for this 3-weekly review visit exacerbated their financial difficulties. This is evident in the narrations below:

*“…The cost of transportation from my village to Tamale is high. We come here very often and because I cannot walk, I have to always come with somebody in a chatted taxi which is expensive. As I sit, I don’t work and have no regular source of income, so it is difficult…” (EP007, female, 85 years, breast cancer)*.

*“…Financially it has not been easy. At some points I could not even come to the hospital on the days they asked me to come because I don’t even have the lorry fare to transport myself from Navrongo all the way here [hospital in Tamale] …” (EP010, female, 68 years, cervical cancer).*

One’s inability to co-finance the treatment and its associated cost suggested that treatment could be discontinued or delayed. This is portrayed by a 70-year-old petty trader suffering from breast cancer:

*“…When I reported to the hospital, they prescribed some drugs which were not available, and I had to buy them from outside for the treatment. I was asked to also do some lab tests but I could not do it because I had no money, hence, I went home without the treatment. Travel cost to Tamale (cancer center) is also high and I have to always come with someone every 3 weeks. Looking at it, I had no option than to wait until I get the money, so I delayed the treatment for some months before coming back.” (EP013, female, 70 years, breast cancer).*

Besides transportation costs, diagnostic or imaging procedures including laboratory tests, X-Rays and dressing of cancer wounds further heightened the financial burden on participants. These are illustrated below:

*“…See all these laboratory test and scans they made to do at Navrongo, they were very expensive. The disease has brought financial burden on me” (EP002, female, 65 years, cervical cancer)*.

### Major theme # 3: Healthcare system strains

Almost all participants in this study talked about health system strains and related them to unfavourable experiences with providers during their diagnosis and treatment processes. Sub-themes identified include negative and insensitive communication and delays in diagnosis and treatment.

#### Negative attitude and insensitive communication

This theme describes various perceived negative attitudes health providers portrayed towards participants in this study during their diagnosis and treatment. Participants complained of disrespectful and abusive care as well as the insensitive way some providers (including cancer specialists) communicated with them and their accompanying family caregivers. In some instances, the provider displayed anger and talked rudely to participants when they are approached about chemotherapy administration. These are illustrated in the narrative below:

*“…some providers will not even listen to you when you are registering a concern. You know we do return travel from far for the treatment. We get here early in the morning by 6:30 am with the hope of returning home early. Normally, after consultation, the doctor will tell you to go for the injections (chemotherapy) which sometimes take up to 3hours of administration. However, the nurses will delay and keep you waiting for hours and when you remind them of the chemo, they get angry and talk harshly to you as if you are a small child. They just disrespect you” (EP009, female, 70 years, cervical cancer)*.

The lack of patience, lack of respect, and absence of comforting and encouraging words from health providers were also identified by participants as healthcare system strain, they encountered. This is evident in the narration below:

*“…As I said earlier if they give you a date and you don’t come on that date, they get angry, but they forget that we need money to come… They should be patient. They just give me a prescription for medicine without adding anything. Some will not will even talk to you to encourage or comfort you. They just want to give you medicine and go away.” (EP010, female, 68 years, cervical cancer)*.

Some of the participants complained of not being directly involved in their treatment decisions. They felt left out of the treatment plan and complained that no provider had open conversations with them regarding their diagnosis, stage of the disease, treatment goals, and recovery. They wondered why physicians preferred to discuss issues regarding their disease conditions and treatment with their children and other family caregivers who accompany them to the hospital other than them. This is reflected in the expressions below:

*“It’s like when they diagnosed it, they do not discuss anything with you the patient, they just barrel you into this protocol and you don’t have the opportunity to ask questions about it and the treatment plan, no time to think…they only talk to the one accompanying you to the hospital but not to you the adult patient. Even now I still have plenty of questions…” (EP005, Female, 64 years, breast cancer)*.

*“No, they didn’t tell me anything. The test too, they don’t tell me anything, as to why I have been put on injections, I do not know. I am not involved in the decisions. They just short you when you try to ask.” (EP003, female, 64 years, cervical cancer)*.

Participants further complained about the insensitive manner providers communicated with them. To them, providers used inappropriate words loosely that constantly reminded them of their impending death. Especially, when they report their pains and other distressing symptoms to them. This is illustrated in the quotes below:

*“I had pains and difficulty swallowing so I complained to the nurse…. she laughed and asked me to draw closer to God… That I should not worry even if I die because I have already enjoyed all the good food in the world…, now I have cancer so anything can happen.” (EP004, Male, 69 years*, Hepatocellular cancer*)*.

#### Delay in diagnosis

This theme describes how participants felt the strain of delays in getting diagnoses or test results, and the strain of having inadequate time to make complex medical decisions. All the participants complained about the lack of competence and skills among health providers in their local facilities (not the highly valued cancer specialists) who kept them for multiple reviews, repetitive diagnostic tests, misdiagnosis, and mistreatment before referring them to a tertiary-level facility for specialist care. Some of this is related to delayed diagnosis:

*“So, I kind of blame my original doctor at the local clinic for not picking up the signs earlier and for poor follow-up care. I reported to that clinic early, but the doctor was treating me with the wrong drugs and giving me dates to report back. He wasted all the time…” (EP006, female, 60 years, cervical cancer)*.

#### Lack of geriatric oncology care

The study found that health providers who provided care to participants were general health providers and nurses who do not have training in caring for elderly patients with cancers. This resulted in participants being managed like the general population with minimal health conditions. This they explained as:

*“The workers [nurses and doctors] are just like those who take care of me when I visit the general clinic. You know our condition, coupled with old age requires much attention… As elderly patients, it will be better we are attended to by professionals trained on our condition [cancer].” (EP009, female, 70 years, cervical cancer)*.

*“The doctors and nurses at the center are not specially trained for cancer and elderly care. You can see that…. You see, it is different when a specialist attends to you! For this, I am treated like any other patient at the general clinic (EP017, Male, 73 years, Prostate cancer)*.

#### Lack of community-based specialist cancer center and long travel distance to access care

Health facilities within the communities of participants were low level health facilities (i.e., health centers and CHPS compounds) that do not have cancer units. Due to their geographical location, participants had to travel long distances and hours to be able to access specialist cancer services, thus, posing geographical barriers in accessing healthcare. These health system barriers were disclosed by participants below:

*“I have to be travelling long distances and hours to the Tamale hospital to access care for my condition.”* (EP004, male, 69 years, Throat cancer).

*“…Each time, I have to travel for hours to the Tamale hospital or other city centers that have cancer centers and doctors to be able to access care…in my community, there is no such facilities or specialists to attend to us.”* (EP020, female, 67 years, breast cancer).

#### Limited availability of essential cancer medicines and other radiation services

According to participants, even after travelling to city centers with the needed cancer facilities and specialists, they were faced with the problem of unavailable or insufficient medications since these facilities could not provide them with the medications to manage their conditions:

*“I am not able to get all the medications for my injections [chemotherapy] most time I come. I always have to go from pharmacy to pharmacy searching for those medications…” (EP016, female, 63 years, Choriocarcinoma)*.

*“When I come here, there has never been an instance where they will say that they have the medications I need available. Always there is a shortage of my medications…” (EP011, female, 67 years, cervical cancer)*.

Besides, depending on the nature and stage of participants’ cancer conditions, a combination of treatments involving chemotherapy, surgery and radiation therapy were used to manage participants’ conditions. Although participants can access the surgery and chemotherapy from the Tamale Teaching Hospital, the facility lacks radiotherapy facilities. Hence, they are referred to other tertiary facilities in other regions to be able to access these treatment services:

“*…one of the issues is that this place they do not have all the treatment that I am supposed to undergo. The radiotherapy for instance, they said they do not have the machine to do it for me. So, the doctor referred me to the Komfo Anokye Teaching Hospital. That is where I go for that treatment all the time. I have to travel to the place from here [this region] …” (EP001, Male, 61 years, Hepatocellular)*.

This also comes with its own challenges since they had to travel to these distant regions, with no relatives to host them. Their unfamiliarity with these geographical settings and health systems imposes some difficulties on them such as increased travelling and accommodation costs for themselves and their caregivers. This was shared:

*“…travelling from the north to Accra is not an easy journey and the cost involved too. The sad part is that when I get there with my daughter, we have to look for a place to sleep. We do not have any family members there. Sometimes we have to even sleep at the hospital opens paces since our money cannot afford a hotel throughout the treatment period and at the same time cater for the treatment costs. Hmmm…”* (EP007, female, 85 years, breast cancer).

### Resources and coping strategies

Participants in the study were able to identify many positive things and characteristics of people that helped them in dealing with the many strains associated with the cancer experience. These positive factors associated with the cancer experience are conceptualized as resources and coping strategies. Major themes identified are: personal resources, family resources, community resources, and healthcare systems resources.

### Major theme #1: personal resources

This theme describes how participants relied on their innate resources including religious beliefs and psychological resources to cope with the experiences and challenges their conditions and treatment had subjected them to. Sub-themes identified included religious beliefs and accepting, adapting to one’s condition, and self-reliance.

#### Religious beliefs

Beliefs were frequently mentioned as helpful by most of our participants. Many Participants questioned why they got cancer and sought for the strength to accept the uncertainty and challenges they faced, turned to or reconnected with their belief in a higher power. It is part of the process of searching for meaning in a situation that defies their understanding. Participants expressed their religious beliefs in the form of giving alms, having faith and belief in God, and surrendering their condition to God through prayers. These were identified as significant personal resources adopted by participants to cope with the strains associated with the cancer experiences. This is evident in the quotes below:

*“But I believe in God so that keeps me going. In my life, I have been through a lot of difficulties and diseases, but God has always delivered me, and I know he will deliver me this time around too. Yea, yea … I know God has a purpose for me.” (EP004, Male, 69 years, Throat cancer)*.

Another reiterated the resourceful role of her religious beliefs:

*“…I was already bleeding so I was praying that God should help the doctors to see the problem so that they will give me medications so that I will be free…” (EP012, female 63 years, cervical cancer)*.

#### Accepting, adapting to one’s condition, and self-reliance

This theme describes personal psychological resources or characteristics developed and used by participants to cope with their condition. To address side effects, and psychological and emotional strains experienced, participants adjusted to their conditions by accepting and learning to stay with them. This strategy was summarised by EP001, a 61-year-old male Hepatocellular cancer patient: *“I have learned to stay with it.” (EP001)*.

Being aware of the gravity and implications of having a cancer, participants described how they employed mental, emotional, and behavioural changes to adapt to the situation

*“…With the changing of the sheets now I do it myself…Initially as I said everything was done for me. Now, most of these things [washing and changing of bed sheets] I always try to do it myself, am not ready to give up now…” (EP004, Male, 69 years, Throat cancer)*.

### Major theme # 2: family resources and support

Various resources, and support obtained from family members are described under this theme. Sub-themes identified comprised: spiritual support, psychological and emotional support, practical support, financial resources, and support.

#### Spiritual support

Spiritual beliefs are used as a coping resource. This was reinforced by participants’ families. Family member as disclosed by participants strengthened their religious beliefs and spirituality through prayers and emphasised on believing in God for recovery. This was shared by participants who clarified the spiritual support they received from their families:

*“Mmm, they say that things will get better I should believe in God and pray, that one day I will be fine again. Even when the sickness started, we [patient and family] did saraka [alms giving]” (EP001, Male, 61 years, Hepatocellular)*.

*“All those taking care of me at home I will say are doing well. They pray for me and support me spiritually.” (EP009, female, 70 years, cervical cancer)*.

#### Psychological and emotional support

This theme portrays how participants with cancer were supported by their family members to cope with the psychological and emotional strains they experienced due to their diagnosis and treatment. Participants explained that their family members acted as a major source of psychological and emotional resources that helped them to cope with their disease. Here, family members encouraged, and shared the participant’s sadness. These were described in the comments below:

*“…They [my family] also give me encouragement and reassurance that I will be fine.” (EP003, female, 64 years, cervical cancer)*.

*“…They show me love and even encourage me…., how they come to sit by me and talk to me is what is still giving me hope to move on.” (EP010, female, 68 years, cervical cancer)*.

#### Practical support with activities of daily living/instrumental activities of daily living

This theme describes the support provided by family members to help participants cope with their inability to perform their daily living activities and instrumental activities of daily living. Due to participants’ lack of functional ability which limited their ability to perform both ADLs and IADLs, family members assumed the role of performing these essential activities of daily living to help participants have a quality life. These roles include assisting participants in bath, grooming themselves, and aiding with cooking to ensure they eat. One of the participants described how his siblings and relatives helped him in performing these roles.

*“… my sister and people are always around to provide support for all practical activities such as washing, cleaning, and cooking. So I don’t do anything.” (EP004, Male, 69 years, Throat cancer)*.

Another participant recounted how their children and grandchildren aided them with their activities of daily living. They explained as follows:

*“…They also cook for me to eat, wash my things when they are dirty and also support me in all my other daily activities. My children and my brothers have been so helpful…” (EP009, female, 70 years, cervical cancer)*.

#### Financial resources and support

Though most participants complained about the heightened cost of treatment for their disease, family members acted as a major source of financial resources for their medical care and cost of living. EP009 for instance revealed that her children catered for all the costs associated with her diagnosis and treatment though it has not been an easy task:

*“Now as am sitting here it is my children who are supporting me bit by bit, they have to pay for the laboratory investigations, the treatment, the cost of my transportation, and the person who will accompany me here. They are doing their best.” (EP018, female, 66 years, cervical)*.

*“It is my children who are helping to raise money to pay for the labs and other things. I have received so much financial support and I have never lacked anything. If these supports were not available, I would have been dead by now…They have been very supportive, they are very good unless I don’t cough [ask or request]. When I cough [when I ask], they will provide…” (EP004, Male, 69 years, Throat cancer)*.

### Major theme # 3: community resources and support

Participants in this study revealed that support from the community provided them with useful resources to help them cope with their strains. They were provided monetary support, were visited, and assisted with tasks. Support from the community and support from the church were sub-themes that emerged.

#### Financial and instrumental support from community members

Forms of support provided by community members and religious bodies are described under this theme. This results in trust among members, and this leads to an increased willingness to engage in helping behaviours. These helping behaviours could include comfort, emotional support, and instrumental support including financial resources. Yet, in most instances, those who receive such helping behaviours return the favour. Primarily, participants explained that they received financial support, visits, and assistance from friends, neighbours, religious institutions, and their social clubs to manage the strains faced due to their treatment. One of the participants in this study revealed the support he received from his friends:

*“They [friends] help me a lot because this my sickness is about a year now and they support me a lot. They give me money; they contribute money, and they also visit.” (EP001, male, 61 years, Hepatocellular)*.

With the mutual dependence and intimate familiarity among rural dwellers, the basis for reciprocity was identified. Helping others within the community creates the foundation for others to extend similar help in times of need as evidenced here:

*“They are doing well; I was a cheerful giver, so my neighbours and friends express the same attitude to me. Some of my neighbours especially those I am in the same group with come around, and they send their contributions very often.” (EP004, male, 69 years, Throat cancer)*.

The instrumental support, particularly monetary support and visitations by a social club comprising market women was specified by a participant who is a member of the club. She indicated:

*“…As a market woman I am a member of the women group in the market so when I was taken ill, they contributed money to come and support me. They also come to visit me from time to time.” (EP012, female, 63 years, cervical cancer)*.

Another participant who was faced with financial strains to get her treatment commence after visiting several health facilities to get diagnosed with cancer explained how a health worker helped her by prosocial assisting her to finance her treatment through an NGO foundation. This is what she had to say:

*“Luckily when I came here and my money finished, one of the doctors [Dr. A.M] added me to a certain foundation that helps old people and they having been paying for my treatment anytime I come.” (EP002, female, 65 years, cervical cancer)*.

### Major theme # 4: Healthcare system resources

While participants noted strains that emanated from the healthcare system, they explained how the healthcare system facilitated access to resources to help them cope with their strains. Only one theme emerged from the data: health support/assistance.

#### Follow-up health support/ assistance

It was found from the study that though the healthcare system posed numerous strains to participants, some characteristics of the healthcare system served as a resource for participants to cope with their health-related strains. This was disclosed by a female participant in this study:

*“…I have one doctor’s number. He gave me his number to call him whenever I face any health issue after attending to me. So I have been calling to complain when am having any problem regarding the disease sometimes he will pick and direct me on what to do or encourage me to come to the hospital…” (EP010, female, 68 years, cervical cancer)*.

## Discussion

The study explores the strains associated with being diagnosed with cancer and the related treatments as well as resources and coping strategies used to address these strains among elderly patients in a rural setting in Ghana. The results from this study revealed that cancer diagnosis and treatment exposed persons living with cancer to side effects including diarrhoea, nausea, loss of appetite, and body weakness due to the chemotherapy and medications which in turn made them appear physically lean compared to their previous weights, discoloured skin, and skin peeling off. This aligns with a previous study by Invernizzi et al. [26] who reported that *“pharmacotherapies and intrinsic tumour-related factors may lead to a wide spectrum of treatment-related disabling complications, such as breast cancer-related lymphedema, axillary web syndrome, persistent pain, bone loss, arthralgia, and fatigue”*. Consistent with previous studies [26–28] our study found that elderly patients experienced severe pains characterised by burning sensations and abdominal cramps, due to the disease and treatment they are undergoing. Though analgesics are prescribed for elderly cancer patients, ageing alters their physiology that reduces the metabolism, absorption, distribution, and purging of pharmacological palliatives [28, 29]. This creates the need for a tailored approach to adequately control and manage pains experienced by this cohort of cancer patients based on their geriatric specificities [30]. Accordingly, symptomatic characteristics of the disease and treatment toxicity including bodily weakness and pains, impaired the ability of these elderly patients to remain functional in terms of performing ADLs and IADLs because pain reduced physical or muscle strength and endurance. The pain intensity and severity experienced by the elderly limit their physical activity and functioning that threatens their health-associated quality of life, life expectancy, and capacity to independently live in a society [31–33].

While other scholars attributed psychological and emotional consequences of cancer disease and treatment to unrelieved pain, their treatment decisions and prognosis, and ageing process [34, 35] our findings suggested that uncertainty, poor treatment outcomes or recovery, fear of death, side effects from therapeutic procedures, cost of care, care-related expenses and inability to finance treatments including hospital stays, and transportation were crucial factors. This aligns with a previous study [36] that found the significance of high existential distress on poor quality of life of persons living with cancer. Compared to developed countries, this resource-deprived rural setting in Ghana lacks well-structured health facilities that provide treatment options and reduce outcome uncertainties as well as economic or welfare systems to offer financial support throughout diagnosis and treatment, particularly for the aged. In this rural setting, health services are provided through health centres and Community-based Health Planning and Services (CHPS) compounds [37, 38] that lack oncology units, experts, and facilities to diagnose and treat cancer diseases. Besides, the National Health Insurance Scheme (NHIS) aimed at eliminating cost barrier to healthcare access, only exempts the elderly aged 70 years and above from paying premiums and covers breast and cervical cancers only [35–37] which does not absorb diagnostics and treatment costs. This led to increased financial burden and subjective financial toxicity among patients due to frequent travels to city centres to access oncology specialists, units and facilities, and co-payment despite not being economically active, which sometimes delayed and halted the treatment process till funds were obtained by these elderly patients. This could deteriorate their life quality meaningfully and increase their mortality. These findings underscore the need for the NHIS and the three-tier pension scheme to respectively cover the cost of care in part or fully and help informal sector workers plan towards end-of-life for the elderly.

Elderly patients’ physical immobility due to their disease limited social interactions and participation in activities of sociocultural significance in their rural communities, leading to some being withdrawn from the groups, which could reduce treatment outcomes because such social circles could enhance illness-management behaviours and treatment adherence [39]. Moreover, the participants acknowledged that experiencing health system strains where health providers portrayed disrespectful, impatient attitudes, lack of encouraging and discomforting words, worsened by insensitive communication, not being directly involved in treatment decisions, as well as delay in diagnosis. Nukpezah et al. [40], however, reported that nurses globally are well-informed and play a crucial role in cancer treatment modalities. This difference in findings might be due to the limited number of nurses with training in oncology and palliative care skills in Ghana. Also, the absence of shared decision concerning treatment could limit full geriatric assessment and reduces the patient-centeredness of care which is important for elderly patients involved with multiple clinicians [41, 42] as in this study. These findings highlight the need for trained oncology and palliative nurses, a collaborative model of care, and communication interventions designed for elderly cancer patients.

Elderly patients were found to rely on personal, family support and community resources to cope with strains experienced from their disease and treatment. Personal religious beliefs acted a source of positive reflections and thoughts that enhanced their perseverance in the face of their strains as found by Caplan et al. [43] and Kahana et al. [44]. This supports the fact that the Ghanaian, especially religious folks are inseparable from their religion since it defines their thoughts and actions [45]. The elderly patients in this study were also able to garner instrumental coping resources including assistance with ADLs/IADLs, financial support, spiritual, and emotion-focused strategies from their families and community members. However, in rural areas, it is worth noting that intimate familiarity (near closeness to each other) and reciprocity (extending support to others in times of difficulties) [27] underpins instrumental support portrayed by community members as identified towards elderly patients in this study. Problem-focused coping strategy where elderly cancer patients were assisted with some health workers to help cope with disease-related symptoms experienced at home is also featured in the current study.

### Strengths and limitations of the study

As a study that employed an exploratory design, the study was able to provide an in-depth exploration of the strains, resources and coping strategies of elderly patients diagnosed with cancer. Also, the study adopted appropriate methodologies to arrive at the findings. Nevertheless, there are some limitations that must be considered. The study was limited to only one specialised hospital. Therefore, we cannot assume that their experiences necessarily reflect all elderly patients who have been diagnosed of cancer because the study did not capture the views of those who were not accessing the health facility. Also, the family caregivers of the patients were not included in the study even though they play an essential role in the journey of the elderly patient diagnosed with cancer. There is also the likelihood of recall bias since this is a self-reported study.

## Conclusion

This study highlights that elderly cancer patients in rural areas in Ghana faced numerous strains associated with being diagnosed with cancer and treatment procedures. These elderly cohorts faced physical, economic, psychological and emotional strains that threatens health and wellbeing. However, they can leverage family, community and health system related resources to navigate through the strains. The study has implications for policy and cancer care for the elderly in rural settings. First, a need to expand advanced health facilities with geriatric oncology units and specialists to improve access to cancer care in rural areas. This may potentially reduce the high-cost burden associated with travelling to access diagnosis and treatment. Also, a tailored approach to effective pain control and management among elderly cancer patients informed by full geriatric assessment is required. Involving elderly patients and their families in treatment decisions to improve the person-centred approach to cancer diagnosis and treatment is also paramount. The results from this study offer underlying reasons for trained oncology and palliative nurses, a collaborative model of care, and communication interventions designed for elderly cancer patients. Finally, the government must assist elderly persons with costs associated with their diagnosis and treatment through the expansion of the NHIS to include this as part of the benefit package.

### Electronic supplementary material

Below is the link to the electronic supplementary material.

Supplementary Material 1

Supplementary Material 2

## Data Availability

The datasets generated and/or analysed during the current study are not publicly available due ethical reasons but are available from the corresponding author on reasonable request.

## List of abbreviations

ADL: Activities of daily living
CHPS: Community-based Health Planning and Services
CHPRE: Committee on Human Research, Publication, and Ethics
IADL: Instrumental activities of daily living
NHIS: National Health Insurance Scheme
