# Supplementary material for: Impact of cancer diagnosis and treatment: a qualitative analysis of strains, resources and coping strategies among elderly patients in a rural setting in Ghana
Source: BMC Geriatr. 2023 Sep 5;23:540. doi: 10.1186/s12877-023-04248-8 (PMC10481500; doi:10.1186/s12877-023-04248-8)
Supplement: Supplementary file 1 — Supplementary Material 1 [file 12877_2023_4248_MOESM1_ESM.docx]

**INTERVIEW GUIDE**

This study aims to explore **the experiences of older adults diagnosed with cancer at the Tamale Teaching Hospital**. The outcome of this study will help policy makers and care providers meet the care needs of older adults diagnosed with cancer. Participation in this study is voluntary and respondent can choose to opt out of the study at any point in time in the course of the study. Again, information provided will be treated as strictly confidential and, in any case, if information will have to be shared with anyone outside the research team, the respondent’s permission will be sought. This interview is expected to last between 30 to 45 minutes.

**Part I: Demographic characteristics**

1. How old are you?
2. What is your sex?
3. What is your main occupation?
4. What is your marital status?
5. What is your religious affiliation?
6. What ethnic group do you belong?
7. What type of cancer have you been diagnosed with?
8. How long have you been living with cancer?

**Guiding Interview Questions**

1. Can you please share with me how aging has affected you as a person?

**Probes:**

1. (ADL, social life, economic life, your health [any co-morbidities such as diabetes, hypertension, heart disease, and its effects on you])
2. Physiological changes such bowel changes, respiratory issues, sight,
3. Please share with me your experience of being diagnosed with cancer.
4. Tell me how the presence of the disease (cancer) has affected your life as a person.

**Probes:**

1. Your Activities Daily Living (ADL’s or self-care activities) such as bathing, grooming, eating, cooking, laundry.
2. Physical life such as mobility (walking), going to the market.
3. Can you please share with me on how the treatment of the disease has affected your life as a person?

**Probes:**

1. Your Activities Daily Living (ADL’s or self-care activities) such as bathing, grooming, eating, cooking, laundry
2. Physical life such as mobility (walking), going to the market
3. Physiologically such as respiratory issues, bowel changes, weight changes
4. Psychologically such as how has it affected you in terms of your emotions (anxiety and depression), body image and self-worth?
5. Financially such as reduction in your economic resources, cost of purchasing treatment,
6. Share with me the things you think you need help with on your health?

**Probes:**

1. Regarding the disease, its treatment, side effects, and symptoms management, laboratory investigations, informational
2. Practical concerns such as assistance with housework
3. Management of cost of disease and treatment.
4. Tell your experience with professional caregivers since your cancer diagnosis and treatment.

**Probes:**

1. Practical support such as home visits, calls to check up on you, were you introduced to a psychologist, any prescribed interventions, and education on what to eat and not eat?
2. Support received regarding symptom management e.g., pain, fatigue, wound, swelling/lumps etc.
3. Assistance to acquire financial support.
4. Tell me about experience with your caregivers at home since your cancer diagnosis and treatment

**Probes:**

1. Attitude of care provider at home.
2. Emotional support such as reassurance, show of love and the feeling of belongingness.
3. Practical help with activities of daily living
4. Social support from family members and other social groupings you belong to.
5. Support with symptom management at home eg pain, fatigue, wound etc
6. Financial support to manage the condition and its treatment
7. Is there any experience you want to add?

**Thank you for taking the time to speak with me today.**
