# Supplementary material for: Impact of cancer diagnosis and treatment: a qualitative analysis of strains, resources and coping strategies among elderly patients in a rural setting in Ghana
Source: BMC Geriatr. 2023 Sep 5;23:540. doi: 10.1186/s12877-023-04248-8 (PMC10481500; doi:10.1186/s12877-023-04248-8)
Supplement: Supplementary file 2 — Supplementary Material 2 [file 12877_2023_4248_MOESM2_ESM.docx]

**Supplementary file 2: demographic characteristics of participants**

| **Participant ID** | **Age**  **(in years)** | **Sex** | **Occupation** | **Cancer type** | **Duration** | **Marital status** | **Religion** | **Ethnicity** |
| --- | --- | --- | --- | --- | --- | --- | --- | --- |
| EP001 | 61 | Male | Businessman | Hepatocellular | 9 months | Married | Muslim | Dagomba |
| EP002 | 65 | Female | Unemployed | Cervical | 1 year | Divorced | Muslim | Dagomba |
| EP003 | 64 | Female | Farmer | Cervical | 3 months | Widow | Christian | Asante |
| EP004 | 69 | Male | Businessman | Throat | 1 year | Divorced | Christian | Kassena |
| EP005 | 64 | Female | Farmer | Breast | 5 months | Widow | Christian | Builsa |
| EP006 | 60 | Female | Trader | Cervical | 6 months | Married | Christian | Kassena |
| EP007 | 85 | Female | Businesswoman | Breast | 2 years | Widow | Muslim | Hausa |
| EP008 | 80 | Female | Retired cleaner | Breast | 5 months | Widow | Christian | Nankam |
| EP009 | 70 | Female | Farmer | Cervical | 2 years | Widow | Muslim | Kusasi |
| EP010 | 68 | Female | Farmer | Cervical | 2 years | Married | Christian | Kassena |
| EP011 | 67 | Female | Unemployed | Cervical | 1 year 6 months | Widow | Muslim | Dagomba |
| EP012 | 63 | Female | Businesswoman | Cervical | 1 year | Divorced | Muslim | Mosi |
| EP013 | 70 | Female | Trader | Breast | 2 years | Married | Muslim | Frafra |
| EP014 | 75 | Female | Unemployed | Breast | 1 year | Widow | Muslim | Dagomba |
| EP015 | 69 | Female | Unemployed | Breast | 2 years | Divorced | Christian | Dagomba |
| EP016 | 63 | Female | Retired teacher | Choriocarcinoma | 1 year | Married | Christian | Mamprusi |
| EP017 | 73 | Male | Retired lab technician | Prostate | 10 months | Married | Traditionalist | Frafra |
| EP018 | 66 | Female | Businesswoman | Cervical | 1 year | Widow | Muslim | Dagomba |
| EP019 | 72 | Female | Unemployed | Breast | 2 years | Widow | Muslim | Kassena |
| EP020 | 67 | Female | Petty trader | Breast | 3 years | Divorced | Christian | Gonja |
